# Supplementary material for: Optimal minimal residual disease threshold in pediatric acute myeloid leukemia: A retrospective cohort study based on the TARGET database
Source: PLoS Med. 2026 May 8;23(5):e1005088. doi: 10.1371/journal.pmed.1005088 (PMC13155632; doi:10.1371/journal.pmed.1005088)
Supplement: S1 Code — (ZIP) [file pmed.1005088.s002.zip › S2 code/PROJ8_5_tbl1/PROJ8_5_tbl1.htm]

## Kaplan Meier Survival Curve

Outcome: First Event
Time: EFS(years)
Landmark analysis for time segment: EFS(years) <= 5
Log rank test: implements the G-rho family of Harrington and Fleming (1982), with weights on each death of S(t)^rho, where S is the Kaplan-Meier estimate of survival. With rho = 0 this is the log-rank or Mantel-Haenszel test.

|  |  |  |  |
| --- | --- | --- | --- |
|  | N | Observed | Expected |
| factor(X23.CS)=0 | 906 | 378 | 494.9954 |
| factor(X23.CS)=1 | 299 | 225 | 108.0046 |

Chisq=
156.0215
on
1
degree of freedom, p=
<0.0001
Survival table

|  |  |  |  |  |  |  |  |
| --- | --- | --- | --- | --- | --- | --- | --- |
|  | EFS(years) | N.Risk | N.Event | N.Censor | Survival | 95%CI Low | 95%CI Upp |
| factor(X23.CS)=0 | 0.16 | 906 | 1 | 0 | 0.9989 | 0.9967 | 1.0000 |
| factor(X23.CS)=0 | 0.17 | 905 | 3 | 0 | 0.9956 | 0.9913 | 0.9999 |
| factor(X23.CS)=0 | 0.18 | 902 | 2 | 0 | 0.9934 | 0.9881 | 0.9987 |
| factor(X23.CS)=0 | 0.19 | 900 | 1 | 1 | 0.9923 | 0.9866 | 0.9980 |
| factor(X23.CS)=0 | 0.2 | 898 | 4 | 0 | 0.9879 | 0.9807 | 0.9950 |
| factor(X23.CS)=0 | 0.21 | 894 | 4 | 0 | 0.9834 | 0.9752 | 0.9918 |
| factor(X23.CS)=0 | 0.22 | 890 | 1 | 0 | 0.9823 | 0.9738 | 0.9909 |
| factor(X23.CS)=0 | 0.23 | 889 | 2 | 1 | 0.9801 | 0.9711 | 0.9893 |
| factor(X23.CS)=0 | 0.24 | 886 | 1 | 0 | 0.9790 | 0.9697 | 0.9884 |
| factor(X23.CS)=0 | 0.26 | 885 | 4 | 0 | 0.9746 | 0.9644 | 0.9849 |
| factor(X23.CS)=0 | 0.27 | 881 | 2 | 1 | 0.9724 | 0.9618 | 0.9831 |
| factor(X23.CS)=0 | 0.28 | 878 | 4 | 0 | 0.9679 | 0.9565 | 0.9795 |
| factor(X23.CS)=0 | 0.29 | 874 | 3 | 0 | 0.9646 | 0.9527 | 0.9767 |
| factor(X23.CS)=0 | 0.31 | 871 | 1 | 0 | 0.9635 | 0.9514 | 0.9758 |
| factor(X23.CS)=0 | 0.32 | 870 | 2 | 0 | 0.9613 | 0.9488 | 0.9740 |
| factor(X23.CS)=0 | 0.33 | 868 | 2 | 0 | 0.9591 | 0.9463 | 0.9721 |
| factor(X23.CS)=0 | 0.37 | 866 | 2 | 0 | 0.9569 | 0.9437 | 0.9702 |
| factor(X23.CS)=0 | 0.38 | 864 | 2 | 0 | 0.9547 | 0.9412 | 0.9683 |
| factor(X23.CS)=0 | 0.4 | 862 | 4 | 0 | 0.9502 | 0.9362 | 0.9645 |
| factor(X23.CS)=0 | 0.41 | 858 | 2 | 0 | 0.9480 | 0.9336 | 0.9626 |
| factor(X23.CS)=0 | 0.42 | 856 | 2 | 0 | 0.9458 | 0.9311 | 0.9607 |
| factor(X23.CS)=0 | 0.44 | 854 | 5 | 0 | 0.9403 | 0.9249 | 0.9558 |
| factor(X23.CS)=0 | 0.45 | 849 | 2 | 0 | 0.9380 | 0.9225 | 0.9539 |
| factor(X23.CS)=0 | 0.46 | 847 | 2 | 0 | 0.9358 | 0.9200 | 0.9519 |
| factor(X23.CS)=0 | 0.47 | 845 | 3 | 0 | 0.9325 | 0.9163 | 0.9490 |
| factor(X23.CS)=0 | 0.5 | 842 | 3 | 0 | 0.9292 | 0.9126 | 0.9461 |
| factor(X23.CS)=0 | 0.51 | 839 | 1 | 0 | 0.9281 | 0.9114 | 0.9451 |
| factor(X23.CS)=0 | 0.52 | 838 | 3 | 0 | 0.9248 | 0.9077 | 0.9421 |
| factor(X23.CS)=0 | 0.53 | 835 | 5 | 0 | 0.9192 | 0.9016 | 0.9372 |
| factor(X23.CS)=0 | 0.54 | 830 | 2 | 0 | 0.9170 | 0.8992 | 0.9352 |
| factor(X23.CS)=0 | 0.55 | 828 | 3 | 0 | 0.9137 | 0.8955 | 0.9322 |
| factor(X23.CS)=0 | 0.56 | 825 | 6 | 0 | 0.9070 | 0.8883 | 0.9262 |
| factor(X23.CS)=0 | 0.57 | 819 | 4 | 0 | 0.9026 | 0.8835 | 0.9221 |
| factor(X23.CS)=0 | 0.58 | 815 | 4 | 0 | 0.8982 | 0.8787 | 0.9181 |
| factor(X23.CS)=0 | 0.59 | 811 | 4 | 0 | 0.8937 | 0.8739 | 0.9141 |
| factor(X23.CS)=0 | 0.6 | 807 | 4 | 0 | 0.8893 | 0.8691 | 0.9100 |
| factor(X23.CS)=0 | 0.61 | 803 | 1 | 0 | 0.8882 | 0.8679 | 0.9090 |
| factor(X23.CS)=0 | 0.62 | 802 | 2 | 0 | 0.8860 | 0.8655 | 0.9070 |
| factor(X23.CS)=0 | 0.63 | 800 | 1 | 0 | 0.8849 | 0.8643 | 0.9059 |
| factor(X23.CS)=0 | 0.64 | 799 | 4 | 0 | 0.8805 | 0.8595 | 0.9019 |
| factor(X23.CS)=0 | 0.65 | 795 | 4 | 0 | 0.8760 | 0.8548 | 0.8978 |
| factor(X23.CS)=0 | 0.66 | 791 | 1 | 0 | 0.8749 | 0.8536 | 0.8968 |
| factor(X23.CS)=0 | 0.67 | 790 | 3 | 0 | 0.8716 | 0.8500 | 0.8937 |
| factor(X23.CS)=0 | 0.68 | 787 | 5 | 0 | 0.8661 | 0.8441 | 0.8886 |
| factor(X23.CS)=0 | 0.69 | 782 | 4 | 0 | 0.8616 | 0.8394 | 0.8844 |
| factor(X23.CS)=0 | 0.7 | 778 | 3 | 0 | 0.8583 | 0.8359 | 0.8814 |
| factor(X23.CS)=0 | 0.71 | 775 | 3 | 0 | 0.8550 | 0.8323 | 0.8783 |
| factor(X23.CS)=0 | 0.72 | 772 | 5 | 0 | 0.8494 | 0.8264 | 0.8731 |
| factor(X23.CS)=0 | 0.73 | 767 | 4 | 0 | 0.8450 | 0.8217 | 0.8689 |
| factor(X23.CS)=0 | 0.74 | 763 | 4 | 0 | 0.8406 | 0.8170 | 0.8648 |
| factor(X23.CS)=0 | 0.75 | 759 | 2 | 0 | 0.8384 | 0.8147 | 0.8627 |
| factor(X23.CS)=0 | 0.76 | 757 | 8 | 0 | 0.8295 | 0.8053 | 0.8544 |
| factor(X23.CS)=0 | 0.77 | 749 | 1 | 0 | 0.8284 | 0.8042 | 0.8534 |
| factor(X23.CS)=0 | 0.78 | 748 | 2 | 0 | 0.8262 | 0.8018 | 0.8513 |
| factor(X23.CS)=0 | 0.79 | 746 | 6 | 0 | 0.8195 | 0.7948 | 0.8450 |
| factor(X23.CS)=0 | 0.8 | 740 | 4 | 0 | 0.8151 | 0.7902 | 0.8408 |
| factor(X23.CS)=0 | 0.81 | 736 | 5 | 0 | 0.8096 | 0.7844 | 0.8356 |
| factor(X23.CS)=0 | 0.82 | 731 | 6 | 0 | 0.8029 | 0.7774 | 0.8293 |
| factor(X23.CS)=0 | 0.83 | 725 | 4 | 0 | 0.7985 | 0.7728 | 0.8251 |
| factor(X23.CS)=0 | 0.84 | 721 | 5 | 0 | 0.7930 | 0.7670 | 0.8198 |
| factor(X23.CS)=0 | 0.85 | 716 | 1 | 0 | 0.7919 | 0.7658 | 0.8188 |
| factor(X23.CS)=0 | 0.86 | 715 | 2 | 0 | 0.7896 | 0.7635 | 0.8167 |
| factor(X23.CS)=0 | 0.87 | 713 | 1 | 0 | 0.7885 | 0.7623 | 0.8156 |
| factor(X23.CS)=0 | 0.88 | 712 | 7 | 0 | 0.7808 | 0.7543 | 0.8082 |
| factor(X23.CS)=0 | 0.89 | 705 | 2 | 0 | 0.7786 | 0.7520 | 0.8061 |
| factor(X23.CS)=0 | 0.9 | 703 | 3 | 0 | 0.7752 | 0.7485 | 0.8029 |
| factor(X23.CS)=0 | 0.91 | 700 | 2 | 0 | 0.7730 | 0.7462 | 0.8008 |
| factor(X23.CS)=0 | 0.92 | 698 | 4 | 0 | 0.7686 | 0.7416 | 0.7966 |
| factor(X23.CS)=0 | 0.93 | 694 | 8 | 0 | 0.7597 | 0.7324 | 0.7881 |
| factor(X23.CS)=0 | 0.94 | 686 | 2 | 0 | 0.7575 | 0.7301 | 0.7860 |
| factor(X23.CS)=0 | 0.95 | 684 | 1 | 0 | 0.7564 | 0.7289 | 0.7849 |
| factor(X23.CS)=0 | 0.96 | 683 | 2 | 1 | 0.7542 | 0.7266 | 0.7828 |
| factor(X23.CS)=0 | 0.97 | 680 | 1 | 0 | 0.7531 | 0.7255 | 0.7817 |
| factor(X23.CS)=0 | 0.99 | 679 | 2 | 1 | 0.7509 | 0.7232 | 0.7796 |
| factor(X23.CS)=0 | 1 | 676 | 4 | 0 | 0.7464 | 0.7186 | 0.7754 |
| factor(X23.CS)=0 | 1.01 | 672 | 3 | 0 | 0.7431 | 0.7151 | 0.7721 |
| factor(X23.CS)=0 | 1.02 | 669 | 3 | 1 | 0.7398 | 0.7117 | 0.7689 |
| factor(X23.CS)=0 | 1.03 | 665 | 3 | 0 | 0.7364 | 0.7082 | 0.7657 |
| factor(X23.CS)=0 | 1.05 | 662 | 6 | 0 | 0.7298 | 0.7014 | 0.7593 |
| factor(X23.CS)=0 | 1.06 | 656 | 2 | 1 | 0.7275 | 0.6991 | 0.7572 |
| factor(X23.CS)=0 | 1.07 | 653 | 4 | 0 | 0.7231 | 0.6945 | 0.7529 |
| factor(X23.CS)=0 | 1.08 | 649 | 3 | 0 | 0.7197 | 0.6910 | 0.7496 |
| factor(X23.CS)=0 | 1.09 | 646 | 1 | 0 | 0.7186 | 0.6899 | 0.7486 |
| factor(X23.CS)=0 | 1.1 | 645 | 2 | 0 | 0.7164 | 0.6876 | 0.7464 |
| factor(X23.CS)=0 | 1.11 | 643 | 0 | 2 | 0.7164 | 0.6876 | 0.7464 |
| factor(X23.CS)=0 | 1.12 | 641 | 3 | 1 | 0.7130 | 0.6841 | 0.7432 |
| factor(X23.CS)=0 | 1.13 | 637 | 4 | 0 | 0.7086 | 0.6795 | 0.7388 |
| factor(X23.CS)=0 | 1.14 | 633 | 2 | 1 | 0.7063 | 0.6772 | 0.7367 |
| factor(X23.CS)=0 | 1.15 | 630 | 1 | 1 | 0.7052 | 0.6761 | 0.7356 |
| factor(X23.CS)=0 | 1.16 | 628 | 1 | 0 | 0.7041 | 0.6749 | 0.7345 |
| factor(X23.CS)=0 | 1.17 | 627 | 1 | 0 | 0.7030 | 0.6738 | 0.7334 |
| factor(X23.CS)=0 | 1.18 | 626 | 1 | 0 | 0.7018 | 0.6726 | 0.7323 |
| factor(X23.CS)=0 | 1.19 | 625 | 2 | 0 | 0.6996 | 0.6703 | 0.7302 |
| factor(X23.CS)=0 | 1.2 | 623 | 3 | 0 | 0.6962 | 0.6668 | 0.7269 |
| factor(X23.CS)=0 | 1.21 | 620 | 1 | 1 | 0.6951 | 0.6657 | 0.7258 |
| factor(X23.CS)=0 | 1.24 | 618 | 1 | 0 | 0.6940 | 0.6645 | 0.7247 |
| factor(X23.CS)=0 | 1.25 | 617 | 0 | 1 | 0.6940 | 0.6645 | 0.7247 |
| factor(X23.CS)=0 | 1.26 | 616 | 1 | 0 | 0.6928 | 0.6634 | 0.7236 |
| factor(X23.CS)=0 | 1.27 | 615 | 4 | 0 | 0.6883 | 0.6587 | 0.7193 |
| factor(X23.CS)=0 | 1.28 | 611 | 2 | 1 | 0.6861 | 0.6564 | 0.7171 |
| factor(X23.CS)=0 | 1.3 | 608 | 3 | 0 | 0.6827 | 0.6530 | 0.7138 |
| factor(X23.CS)=0 | 1.32 | 605 | 1 | 0 | 0.6816 | 0.6518 | 0.7127 |
| factor(X23.CS)=0 | 1.34 | 604 | 3 | 1 | 0.6782 | 0.6483 | 0.7094 |
| factor(X23.CS)=0 | 1.35 | 600 | 1 | 0 | 0.6771 | 0.6472 | 0.7083 |
| factor(X23.CS)=0 | 1.36 | 599 | 2 | 0 | 0.6748 | 0.6449 | 0.7061 |
| factor(X23.CS)=0 | 1.37 | 597 | 2 | 1 | 0.6725 | 0.6426 | 0.7039 |
| factor(X23.CS)=0 | 1.38 | 594 | 2 | 0 | 0.6703 | 0.6402 | 0.7017 |
| factor(X23.CS)=0 | 1.39 | 592 | 2 | 0 | 0.6680 | 0.6379 | 0.6995 |
| factor(X23.CS)=0 | 1.4 | 590 | 1 | 0 | 0.6669 | 0.6368 | 0.6984 |
| factor(X23.CS)=0 | 1.41 | 589 | 1 | 0 | 0.6657 | 0.6356 | 0.6973 |
| factor(X23.CS)=0 | 1.42 | 588 | 4 | 0 | 0.6612 | 0.6310 | 0.6929 |
| factor(X23.CS)=0 | 1.43 | 584 | 4 | 0 | 0.6567 | 0.6264 | 0.6885 |
| factor(X23.CS)=0 | 1.45 | 580 | 1 | 0 | 0.6555 | 0.6252 | 0.6874 |
| factor(X23.CS)=0 | 1.48 | 579 | 2 | 0 | 0.6533 | 0.6229 | 0.6852 |
| factor(X23.CS)=0 | 1.49 | 577 | 3 | 0 | 0.6499 | 0.6194 | 0.6818 |
| factor(X23.CS)=0 | 1.51 | 574 | 1 | 0 | 0.6488 | 0.6183 | 0.6807 |
| factor(X23.CS)=0 | 1.53 | 573 | 1 | 0 | 0.6476 | 0.6171 | 0.6796 |
| factor(X23.CS)=0 | 1.54 | 572 | 1 | 0 | 0.6465 | 0.6160 | 0.6785 |
| factor(X23.CS)=0 | 1.55 | 571 | 1 | 0 | 0.6454 | 0.6148 | 0.6774 |
| factor(X23.CS)=0 | 1.56 | 570 | 1 | 0 | 0.6442 | 0.6137 | 0.6763 |
| factor(X23.CS)=0 | 1.58 | 569 | 4 | 0 | 0.6397 | 0.6090 | 0.6719 |
| factor(X23.CS)=0 | 1.6 | 565 | 2 | 0 | 0.6374 | 0.6067 | 0.6697 |
| factor(X23.CS)=0 | 1.61 | 563 | 0 | 1 | 0.6374 | 0.6067 | 0.6697 |
| factor(X23.CS)=0 | 1.62 | 562 | 2 | 0 | 0.6352 | 0.6044 | 0.6675 |
| factor(X23.CS)=0 | 1.64 | 560 | 1 | 0 | 0.6340 | 0.6033 | 0.6663 |
| factor(X23.CS)=0 | 1.65 | 559 | 2 | 0 | 0.6318 | 0.6010 | 0.6641 |
| factor(X23.CS)=0 | 1.66 | 557 | 1 | 0 | 0.6306 | 0.5998 | 0.6630 |
| factor(X23.CS)=0 | 1.67 | 556 | 1 | 0 | 0.6295 | 0.5987 | 0.6619 |
| factor(X23.CS)=0 | 1.71 | 555 | 0 | 1 | 0.6295 | 0.5987 | 0.6619 |
| factor(X23.CS)=0 | 1.72 | 554 | 2 | 0 | 0.6272 | 0.5964 | 0.6597 |
| factor(X23.CS)=0 | 1.75 | 552 | 0 | 1 | 0.6272 | 0.5964 | 0.6597 |
| factor(X23.CS)=0 | 1.77 | 551 | 1 | 0 | 0.6261 | 0.5952 | 0.6586 |
| factor(X23.CS)=0 | 1.78 | 550 | 1 | 0 | 0.6249 | 0.5940 | 0.6574 |
| factor(X23.CS)=0 | 1.81 | 549 | 1 | 0 | 0.6238 | 0.5929 | 0.6563 |
| factor(X23.CS)=0 | 1.83 | 548 | 0 | 1 | 0.6238 | 0.5929 | 0.6563 |
| factor(X23.CS)=0 | 1.87 | 547 | 1 | 1 | 0.6227 | 0.5917 | 0.6552 |
| factor(X23.CS)=0 | 1.89 | 545 | 2 | 0 | 0.6204 | 0.5894 | 0.6530 |
| factor(X23.CS)=0 | 1.9 | 543 | 2 | 0 | 0.6181 | 0.5871 | 0.6507 |
| factor(X23.CS)=0 | 1.92 | 541 | 1 | 1 | 0.6170 | 0.5859 | 0.6496 |
| factor(X23.CS)=0 | 1.94 | 539 | 1 | 2 | 0.6158 | 0.5848 | 0.6485 |
| factor(X23.CS)=0 | 1.96 | 536 | 0 | 1 | 0.6158 | 0.5848 | 0.6485 |
| factor(X23.CS)=0 | 1.98 | 535 | 0 | 1 | 0.6158 | 0.5848 | 0.6485 |
| factor(X23.CS)=0 | 1.99 | 534 | 1 | 0 | 0.6147 | 0.5836 | 0.6474 |
| factor(X23.CS)=0 | 2.03 | 533 | 1 | 0 | 0.6135 | 0.5824 | 0.6462 |
| factor(X23.CS)=0 | 2.05 | 532 | 1 | 0 | 0.6123 | 0.5813 | 0.6451 |
| factor(X23.CS)=0 | 2.07 | 531 | 1 | 0 | 0.6112 | 0.5801 | 0.6440 |
| factor(X23.CS)=0 | 2.08 | 530 | 1 | 0 | 0.6100 | 0.5789 | 0.6428 |
| factor(X23.CS)=0 | 2.09 | 529 | 1 | 0 | 0.6089 | 0.5777 | 0.6417 |
| factor(X23.CS)=0 | 2.1 | 528 | 0 | 1 | 0.6089 | 0.5777 | 0.6417 |
| factor(X23.CS)=0 | 2.11 | 527 | 1 | 0 | 0.6077 | 0.5766 | 0.6406 |
| factor(X23.CS)=0 | 2.13 | 526 | 1 | 0 | 0.6066 | 0.5754 | 0.6394 |
| factor(X23.CS)=0 | 2.14 | 525 | 1 | 0 | 0.6054 | 0.5742 | 0.6383 |
| factor(X23.CS)=0 | 2.15 | 524 | 1 | 0 | 0.6043 | 0.5731 | 0.6372 |
| factor(X23.CS)=0 | 2.18 | 523 | 0 | 1 | 0.6043 | 0.5731 | 0.6372 |
| factor(X23.CS)=0 | 2.2 | 522 | 1 | 0 | 0.6031 | 0.5719 | 0.6360 |
| factor(X23.CS)=0 | 2.22 | 521 | 2 | 1 | 0.6008 | 0.5695 | 0.6338 |
| factor(X23.CS)=0 | 2.23 | 518 | 1 | 1 | 0.5996 | 0.5684 | 0.6326 |
| factor(X23.CS)=0 | 2.27 | 516 | 0 | 2 | 0.5996 | 0.5684 | 0.6326 |
| factor(X23.CS)=0 | 2.3 | 514 | 1 | 0 | 0.5985 | 0.5672 | 0.6315 |
| factor(X23.CS)=0 | 2.31 | 513 | 1 | 0 | 0.5973 | 0.5660 | 0.6303 |
| factor(X23.CS)=0 | 2.32 | 512 | 0 | 1 | 0.5973 | 0.5660 | 0.6303 |
| factor(X23.CS)=0 | 2.34 | 511 | 1 | 0 | 0.5961 | 0.5648 | 0.6292 |
| factor(X23.CS)=0 | 2.36 | 510 | 1 | 0 | 0.5950 | 0.5636 | 0.6280 |
| factor(X23.CS)=0 | 2.37 | 509 | 1 | 1 | 0.5938 | 0.5624 | 0.6269 |
| factor(X23.CS)=0 | 2.39 | 507 | 0 | 1 | 0.5938 | 0.5624 | 0.6269 |
| factor(X23.CS)=0 | 2.4 | 506 | 0 | 1 | 0.5938 | 0.5624 | 0.6269 |
| factor(X23.CS)=0 | 2.41 | 505 | 0 | 1 | 0.5938 | 0.5624 | 0.6269 |
| factor(X23.CS)=0 | 2.42 | 504 | 1 | 0 | 0.5926 | 0.5612 | 0.6257 |
| factor(X23.CS)=0 | 2.43 | 503 | 0 | 1 | 0.5926 | 0.5612 | 0.6257 |
| factor(X23.CS)=0 | 2.44 | 502 | 0 | 2 | 0.5926 | 0.5612 | 0.6257 |
| factor(X23.CS)=0 | 2.46 | 500 | 0 | 1 | 0.5926 | 0.5612 | 0.6257 |
| factor(X23.CS)=0 | 2.47 | 499 | 0 | 1 | 0.5926 | 0.5612 | 0.6257 |
| factor(X23.CS)=0 | 2.48 | 498 | 1 | 0 | 0.5914 | 0.5600 | 0.6246 |
| factor(X23.CS)=0 | 2.49 | 497 | 0 | 1 | 0.5914 | 0.5600 | 0.6246 |
| factor(X23.CS)=0 | 2.5 | 496 | 0 | 4 | 0.5914 | 0.5600 | 0.6246 |
| factor(X23.CS)=0 | 2.51 | 492 | 1 | 1 | 0.5902 | 0.5588 | 0.6234 |
| factor(X23.CS)=0 | 2.52 | 490 | 1 | 0 | 0.5890 | 0.5576 | 0.6222 |
| factor(X23.CS)=0 | 2.53 | 489 | 0 | 1 | 0.5890 | 0.5576 | 0.6222 |
| factor(X23.CS)=0 | 2.55 | 488 | 0 | 1 | 0.5890 | 0.5576 | 0.6222 |
| factor(X23.CS)=0 | 2.56 | 487 | 0 | 1 | 0.5890 | 0.5576 | 0.6222 |
| factor(X23.CS)=0 | 2.6 | 486 | 0 | 1 | 0.5890 | 0.5576 | 0.6222 |
| factor(X23.CS)=0 | 2.61 | 485 | 1 | 1 | 0.5878 | 0.5564 | 0.6210 |
| factor(X23.CS)=0 | 2.62 | 483 | 2 | 0 | 0.5854 | 0.5539 | 0.6186 |
| factor(X23.CS)=0 | 2.63 | 481 | 0 | 1 | 0.5854 | 0.5539 | 0.6186 |
| factor(X23.CS)=0 | 2.64 | 480 | 0 | 1 | 0.5854 | 0.5539 | 0.6186 |
| factor(X23.CS)=0 | 2.68 | 479 | 0 | 1 | 0.5854 | 0.5539 | 0.6186 |
| factor(X23.CS)=0 | 2.7 | 478 | 1 | 1 | 0.5841 | 0.5526 | 0.6174 |
| factor(X23.CS)=0 | 2.73 | 476 | 0 | 1 | 0.5841 | 0.5526 | 0.6174 |
| factor(X23.CS)=0 | 2.75 | 475 | 0 | 1 | 0.5841 | 0.5526 | 0.6174 |
| factor(X23.CS)=0 | 2.78 | 474 | 0 | 2 | 0.5841 | 0.5526 | 0.6174 |
| factor(X23.CS)=0 | 2.79 | 472 | 0 | 2 | 0.5841 | 0.5526 | 0.6174 |
| factor(X23.CS)=0 | 2.83 | 470 | 0 | 1 | 0.5841 | 0.5526 | 0.6174 |
| factor(X23.CS)=0 | 2.89 | 469 | 0 | 1 | 0.5841 | 0.5526 | 0.6174 |
| factor(X23.CS)=0 | 2.93 | 468 | 0 | 1 | 0.5841 | 0.5526 | 0.6174 |
| factor(X23.CS)=0 | 2.98 | 467 | 0 | 1 | 0.5841 | 0.5526 | 0.6174 |
| factor(X23.CS)=0 | 3.01 | 466 | 0 | 1 | 0.5841 | 0.5526 | 0.6174 |
| factor(X23.CS)=0 | 3.02 | 465 | 0 | 1 | 0.5841 | 0.5526 | 0.6174 |
| factor(X23.CS)=0 | 3.03 | 464 | 0 | 1 | 0.5841 | 0.5526 | 0.6174 |
| factor(X23.CS)=0 | 3.04 | 463 | 0 | 2 | 0.5841 | 0.5526 | 0.6174 |
| factor(X23.CS)=0 | 3.05 | 461 | 0 | 1 | 0.5841 | 0.5526 | 0.6174 |
| factor(X23.CS)=0 | 3.07 | 460 | 0 | 1 | 0.5841 | 0.5526 | 0.6174 |
| factor(X23.CS)=0 | 3.11 | 459 | 0 | 1 | 0.5841 | 0.5526 | 0.6174 |
| factor(X23.CS)=0 | 3.14 | 458 | 0 | 1 | 0.5841 | 0.5526 | 0.6174 |
| factor(X23.CS)=0 | 3.15 | 457 | 0 | 1 | 0.5841 | 0.5526 | 0.6174 |
| factor(X23.CS)=0 | 3.16 | 456 | 0 | 2 | 0.5841 | 0.5526 | 0.6174 |
| factor(X23.CS)=0 | 3.17 | 454 | 0 | 1 | 0.5841 | 0.5526 | 0.6174 |
| factor(X23.CS)=0 | 3.18 | 453 | 1 | 2 | 0.5829 | 0.5513 | 0.6162 |
| factor(X23.CS)=0 | 3.19 | 450 | 0 | 1 | 0.5829 | 0.5513 | 0.6162 |
| factor(X23.CS)=0 | 3.2 | 449 | 0 | 1 | 0.5829 | 0.5513 | 0.6162 |
| factor(X23.CS)=0 | 3.22 | 448 | 0 | 1 | 0.5829 | 0.5513 | 0.6162 |
| factor(X23.CS)=0 | 3.23 | 447 | 0 | 1 | 0.5829 | 0.5513 | 0.6162 |
| factor(X23.CS)=0 | 3.24 | 446 | 0 | 1 | 0.5829 | 0.5513 | 0.6162 |
| factor(X23.CS)=0 | 3.25 | 445 | 0 | 1 | 0.5829 | 0.5513 | 0.6162 |
| factor(X23.CS)=0 | 3.28 | 444 | 0 | 3 | 0.5829 | 0.5513 | 0.6162 |
| factor(X23.CS)=0 | 3.29 | 441 | 0 | 1 | 0.5829 | 0.5513 | 0.6162 |
| factor(X23.CS)=0 | 3.3 | 440 | 0 | 2 | 0.5829 | 0.5513 | 0.6162 |
| factor(X23.CS)=0 | 3.32 | 438 | 0 | 1 | 0.5829 | 0.5513 | 0.6162 |
| factor(X23.CS)=0 | 3.33 | 437 | 0 | 3 | 0.5829 | 0.5513 | 0.6162 |
| factor(X23.CS)=0 | 3.34 | 434 | 0 | 2 | 0.5829 | 0.5513 | 0.6162 |
| factor(X23.CS)=0 | 3.35 | 432 | 0 | 3 | 0.5829 | 0.5513 | 0.6162 |
| factor(X23.CS)=0 | 3.36 | 429 | 0 | 2 | 0.5829 | 0.5513 | 0.6162 |
| factor(X23.CS)=0 | 3.38 | 427 | 0 | 3 | 0.5829 | 0.5513 | 0.6162 |
| factor(X23.CS)=0 | 3.39 | 424 | 1 | 1 | 0.5815 | 0.5499 | 0.6148 |
| factor(X23.CS)=0 | 3.4 | 422 | 0 | 1 | 0.5815 | 0.5499 | 0.6148 |
| factor(X23.CS)=0 | 3.41 | 421 | 0 | 4 | 0.5815 | 0.5499 | 0.6148 |
| factor(X23.CS)=0 | 3.43 | 417 | 0 | 1 | 0.5815 | 0.5499 | 0.6148 |
| factor(X23.CS)=0 | 3.44 | 416 | 0 | 4 | 0.5815 | 0.5499 | 0.6148 |
| factor(X23.CS)=0 | 3.45 | 412 | 0 | 2 | 0.5815 | 0.5499 | 0.6148 |
| factor(X23.CS)=0 | 3.46 | 410 | 0 | 1 | 0.5815 | 0.5499 | 0.6148 |
| factor(X23.CS)=0 | 3.48 | 409 | 0 | 2 | 0.5815 | 0.5499 | 0.6148 |
| factor(X23.CS)=0 | 3.5 | 407 | 0 | 3 | 0.5815 | 0.5499 | 0.6148 |
| factor(X23.CS)=0 | 3.52 | 404 | 0 | 1 | 0.5815 | 0.5499 | 0.6148 |
| factor(X23.CS)=0 | 3.54 | 403 | 1 | 1 | 0.5800 | 0.5484 | 0.6134 |
| factor(X23.CS)=0 | 3.56 | 401 | 0 | 4 | 0.5800 | 0.5484 | 0.6134 |
| factor(X23.CS)=0 | 3.58 | 397 | 0 | 1 | 0.5800 | 0.5484 | 0.6134 |
| factor(X23.CS)=0 | 3.62 | 396 | 0 | 1 | 0.5800 | 0.5484 | 0.6134 |
| factor(X23.CS)=0 | 3.63 | 395 | 0 | 1 | 0.5800 | 0.5484 | 0.6134 |
| factor(X23.CS)=0 | 3.65 | 394 | 0 | 2 | 0.5800 | 0.5484 | 0.6134 |
| factor(X23.CS)=0 | 3.66 | 392 | 0 | 1 | 0.5800 | 0.5484 | 0.6134 |
| factor(X23.CS)=0 | 3.74 | 391 | 0 | 1 | 0.5800 | 0.5484 | 0.6134 |
| factor(X23.CS)=0 | 3.79 | 390 | 0 | 1 | 0.5800 | 0.5484 | 0.6134 |
| factor(X23.CS)=0 | 3.81 | 389 | 0 | 2 | 0.5800 | 0.5484 | 0.6134 |
| factor(X23.CS)=0 | 3.83 | 387 | 0 | 1 | 0.5800 | 0.5484 | 0.6134 |
| factor(X23.CS)=0 | 3.84 | 386 | 0 | 2 | 0.5800 | 0.5484 | 0.6134 |
| factor(X23.CS)=0 | 3.89 | 384 | 0 | 1 | 0.5800 | 0.5484 | 0.6134 |
| factor(X23.CS)=0 | 3.9 | 383 | 0 | 2 | 0.5800 | 0.5484 | 0.6134 |
| factor(X23.CS)=0 | 3.95 | 381 | 0 | 1 | 0.5800 | 0.5484 | 0.6134 |
| factor(X23.CS)=0 | 3.96 | 380 | 0 | 1 | 0.5800 | 0.5484 | 0.6134 |
| factor(X23.CS)=0 | 3.97 | 379 | 0 | 1 | 0.5800 | 0.5484 | 0.6134 |
| factor(X23.CS)=0 | 3.99 | 378 | 1 | 0 | 0.5785 | 0.5469 | 0.6120 |
| factor(X23.CS)=0 | 4.04 | 377 | 0 | 1 | 0.5785 | 0.5469 | 0.6120 |
| factor(X23.CS)=0 | 4.05 | 376 | 0 | 2 | 0.5785 | 0.5469 | 0.6120 |
| factor(X23.CS)=0 | 4.06 | 374 | 0 | 2 | 0.5785 | 0.5469 | 0.6120 |
| factor(X23.CS)=0 | 4.07 | 372 | 1 | 1 | 0.5769 | 0.5453 | 0.6105 |
| factor(X23.CS)=0 | 4.08 | 370 | 0 | 2 | 0.5769 | 0.5453 | 0.6105 |
| factor(X23.CS)=0 | 4.13 | 368 | 0 | 2 | 0.5769 | 0.5453 | 0.6105 |
| factor(X23.CS)=0 | 4.15 | 366 | 0 | 2 | 0.5769 | 0.5453 | 0.6105 |
| factor(X23.CS)=0 | 4.19 | 364 | 0 | 2 | 0.5769 | 0.5453 | 0.6105 |
| factor(X23.CS)=0 | 4.22 | 362 | 0 | 1 | 0.5769 | 0.5453 | 0.6105 |
| factor(X23.CS)=0 | 4.23 | 361 | 1 | 0 | 0.5754 | 0.5436 | 0.6089 |
| factor(X23.CS)=0 | 4.26 | 360 | 0 | 1 | 0.5754 | 0.5436 | 0.6089 |
| factor(X23.CS)=0 | 4.27 | 359 | 0 | 2 | 0.5754 | 0.5436 | 0.6089 |
| factor(X23.CS)=0 | 4.31 | 357 | 0 | 2 | 0.5754 | 0.5436 | 0.6089 |
| factor(X23.CS)=0 | 4.32 | 355 | 0 | 1 | 0.5754 | 0.5436 | 0.6089 |
| factor(X23.CS)=0 | 4.34 | 354 | 0 | 1 | 0.5754 | 0.5436 | 0.6089 |
| factor(X23.CS)=0 | 4.35 | 353 | 0 | 4 | 0.5754 | 0.5436 | 0.6089 |
| factor(X23.CS)=0 | 4.36 | 349 | 0 | 4 | 0.5754 | 0.5436 | 0.6089 |
| factor(X23.CS)=0 | 4.38 | 345 | 0 | 3 | 0.5754 | 0.5436 | 0.6089 |
| factor(X23.CS)=0 | 4.4 | 342 | 0 | 1 | 0.5754 | 0.5436 | 0.6089 |
| factor(X23.CS)=0 | 4.41 | 341 | 0 | 1 | 0.5754 | 0.5436 | 0.6089 |
| factor(X23.CS)=0 | 4.42 | 340 | 0 | 2 | 0.5754 | 0.5436 | 0.6089 |
| factor(X23.CS)=0 | 4.44 | 338 | 0 | 2 | 0.5754 | 0.5436 | 0.6089 |
| factor(X23.CS)=0 | 4.45 | 336 | 0 | 1 | 0.5754 | 0.5436 | 0.6089 |
| factor(X23.CS)=0 | 4.47 | 335 | 0 | 1 | 0.5754 | 0.5436 | 0.6089 |
| factor(X23.CS)=0 | 4.48 | 334 | 0 | 1 | 0.5754 | 0.5436 | 0.6089 |
| factor(X23.CS)=0 | 4.49 | 333 | 0 | 2 | 0.5754 | 0.5436 | 0.6089 |
| factor(X23.CS)=0 | 4.5 | 331 | 0 | 1 | 0.5754 | 0.5436 | 0.6089 |
| factor(X23.CS)=0 | 4.53 | 330 | 0 | 1 | 0.5754 | 0.5436 | 0.6089 |
| factor(X23.CS)=0 | 4.54 | 329 | 0 | 1 | 0.5754 | 0.5436 | 0.6089 |
| factor(X23.CS)=0 | 4.58 | 328 | 0 | 2 | 0.5754 | 0.5436 | 0.6089 |
| factor(X23.CS)=0 | 4.59 | 326 | 0 | 1 | 0.5754 | 0.5436 | 0.6089 |
| factor(X23.CS)=0 | 4.6 | 325 | 0 | 3 | 0.5754 | 0.5436 | 0.6089 |
| factor(X23.CS)=0 | 4.61 | 322 | 0 | 3 | 0.5754 | 0.5436 | 0.6089 |
| factor(X23.CS)=0 | 4.62 | 319 | 0 | 1 | 0.5754 | 0.5436 | 0.6089 |
| factor(X23.CS)=0 | 4.64 | 318 | 0 | 1 | 0.5754 | 0.5436 | 0.6089 |
| factor(X23.CS)=0 | 4.72 | 317 | 0 | 1 | 0.5754 | 0.5436 | 0.6089 |
| factor(X23.CS)=0 | 4.73 | 316 | 0 | 2 | 0.5754 | 0.5436 | 0.6089 |
| factor(X23.CS)=0 | 4.75 | 314 | 0 | 1 | 0.5754 | 0.5436 | 0.6089 |
| factor(X23.CS)=0 | 4.79 | 313 | 0 | 1 | 0.5754 | 0.5436 | 0.6089 |
| factor(X23.CS)=0 | 4.81 | 312 | 0 | 1 | 0.5754 | 0.5436 | 0.6089 |
| factor(X23.CS)=0 | 4.82 | 311 | 0 | 1 | 0.5754 | 0.5436 | 0.6089 |
| factor(X23.CS)=0 | 4.85 | 310 | 0 | 4 | 0.5754 | 0.5436 | 0.6089 |
| factor(X23.CS)=0 | 4.87 | 306 | 0 | 2 | 0.5754 | 0.5436 | 0.6089 |
| factor(X23.CS)=0 | 4.9 | 304 | 0 | 1 | 0.5754 | 0.5436 | 0.6089 |
| factor(X23.CS)=0 | 4.91 | 303 | 0 | 1 | 0.5754 | 0.5436 | 0.6089 |
| factor(X23.CS)=0 | 4.94 | 302 | 0 | 2 | 0.5754 | 0.5436 | 0.6089 |
| factor(X23.CS)=0 | 4.97 | 300 | 0 | 1 | 0.5754 | 0.5436 | 0.6089 |
| factor(X23.CS)=0 | 4.99 | 299 | 0 | 1 | 0.5754 | 0.5436 | 0.6089 |
| factor(X23.CS)=0 | 5 | 298 | 0 | 298 | 0.5754 | 0.5436 | 0.6089 |
| factor(X23.CS)=1 | 0.13 | 299 | 2 | 0 | 0.9933 | 0.9841 | 1.0000 |
| factor(X23.CS)=1 | 0.15 | 297 | 2 | 0 | 0.9866 | 0.9737 | 0.9997 |
| factor(X23.CS)=1 | 0.16 | 295 | 5 | 0 | 0.9699 | 0.9507 | 0.9895 |
| factor(X23.CS)=1 | 0.17 | 290 | 3 | 0 | 0.9599 | 0.9379 | 0.9824 |
| factor(X23.CS)=1 | 0.18 | 287 | 4 | 0 | 0.9465 | 0.9213 | 0.9723 |
| factor(X23.CS)=1 | 0.19 | 283 | 11 | 0 | 0.9097 | 0.8778 | 0.9428 |
| factor(X23.CS)=1 | 0.2 | 272 | 4 | 0 | 0.8963 | 0.8624 | 0.9315 |
| factor(X23.CS)=1 | 0.21 | 268 | 11 | 0 | 0.8595 | 0.8210 | 0.8998 |
| factor(X23.CS)=1 | 0.22 | 257 | 10 | 0 | 0.8261 | 0.7842 | 0.8702 |
| factor(X23.CS)=1 | 0.23 | 247 | 8 | 0 | 0.7993 | 0.7552 | 0.8460 |
| factor(X23.CS)=1 | 0.24 | 239 | 3 | 0 | 0.7893 | 0.7444 | 0.8369 |
| factor(X23.CS)=1 | 0.25 | 236 | 3 | 0 | 0.7793 | 0.7336 | 0.8277 |
| factor(X23.CS)=1 | 0.27 | 233 | 2 | 0 | 0.7726 | 0.7265 | 0.8216 |
| factor(X23.CS)=1 | 0.28 | 231 | 3 | 0 | 0.7625 | 0.7158 | 0.8123 |
| factor(X23.CS)=1 | 0.29 | 228 | 1 | 0 | 0.7592 | 0.7122 | 0.8092 |
| factor(X23.CS)=1 | 0.3 | 227 | 3 | 0 | 0.7492 | 0.7016 | 0.7999 |
| factor(X23.CS)=1 | 0.32 | 224 | 2 | 0 | 0.7425 | 0.6945 | 0.7937 |
| factor(X23.CS)=1 | 0.33 | 222 | 1 | 1 | 0.7391 | 0.6910 | 0.7906 |
| factor(X23.CS)=1 | 0.34 | 220 | 1 | 0 | 0.7358 | 0.6875 | 0.7875 |
| factor(X23.CS)=1 | 0.35 | 219 | 2 | 0 | 0.7291 | 0.6804 | 0.7812 |
| factor(X23.CS)=1 | 0.36 | 217 | 1 | 0 | 0.7257 | 0.6768 | 0.7781 |
| factor(X23.CS)=1 | 0.37 | 216 | 1 | 0 | 0.7223 | 0.6733 | 0.7749 |
| factor(X23.CS)=1 | 0.41 | 215 | 2 | 0 | 0.7156 | 0.6663 | 0.7686 |
| factor(X23.CS)=1 | 0.42 | 213 | 1 | 0 | 0.7123 | 0.6627 | 0.7655 |
| factor(X23.CS)=1 | 0.43 | 212 | 1 | 0 | 0.7089 | 0.6592 | 0.7623 |
| factor(X23.CS)=1 | 0.45 | 211 | 1 | 0 | 0.7055 | 0.6557 | 0.7592 |
| factor(X23.CS)=1 | 0.47 | 210 | 2 | 0 | 0.6988 | 0.6487 | 0.7528 |
| factor(X23.CS)=1 | 0.48 | 208 | 2 | 0 | 0.6921 | 0.6417 | 0.7465 |
| factor(X23.CS)=1 | 0.49 | 206 | 3 | 0 | 0.6820 | 0.6312 | 0.7369 |
| factor(X23.CS)=1 | 0.5 | 203 | 1 | 0 | 0.6787 | 0.6277 | 0.7337 |
| factor(X23.CS)=1 | 0.52 | 202 | 2 | 0 | 0.6719 | 0.6207 | 0.7274 |
| factor(X23.CS)=1 | 0.53 | 200 | 3 | 0 | 0.6619 | 0.6103 | 0.7178 |
| factor(X23.CS)=1 | 0.54 | 197 | 1 | 0 | 0.6585 | 0.6068 | 0.7145 |
| factor(X23.CS)=1 | 0.55 | 196 | 2 | 0 | 0.6518 | 0.5999 | 0.7081 |
| factor(X23.CS)=1 | 0.56 | 194 | 1 | 0 | 0.6484 | 0.5965 | 0.7049 |
| factor(X23.CS)=1 | 0.57 | 193 | 1 | 0 | 0.6451 | 0.5930 | 0.7017 |
| factor(X23.CS)=1 | 0.58 | 192 | 1 | 0 | 0.6417 | 0.5895 | 0.6985 |
| factor(X23.CS)=1 | 0.59 | 191 | 1 | 0 | 0.6383 | 0.5861 | 0.6952 |
| factor(X23.CS)=1 | 0.6 | 190 | 1 | 0 | 0.6350 | 0.5826 | 0.6920 |
| factor(X23.CS)=1 | 0.62 | 189 | 1 | 0 | 0.6316 | 0.5792 | 0.6888 |
| factor(X23.CS)=1 | 0.64 | 188 | 1 | 0 | 0.6283 | 0.5758 | 0.6856 |
| factor(X23.CS)=1 | 0.66 | 187 | 1 | 0 | 0.6249 | 0.5723 | 0.6823 |
| factor(X23.CS)=1 | 0.68 | 186 | 3 | 0 | 0.6148 | 0.5620 | 0.6726 |
| factor(X23.CS)=1 | 0.69 | 183 | 2 | 0 | 0.6081 | 0.5552 | 0.6661 |
| factor(X23.CS)=1 | 0.7 | 181 | 4 | 0 | 0.5947 | 0.5415 | 0.6531 |
| factor(X23.CS)=1 | 0.71 | 177 | 1 | 0 | 0.5913 | 0.5381 | 0.6498 |
| factor(X23.CS)=1 | 0.72 | 176 | 3 | 0 | 0.5812 | 0.5279 | 0.6400 |
| factor(X23.CS)=1 | 0.74 | 173 | 2 | 0 | 0.5745 | 0.5211 | 0.6334 |
| factor(X23.CS)=1 | 0.75 | 171 | 2 | 0 | 0.5678 | 0.5143 | 0.6269 |
| factor(X23.CS)=1 | 0.76 | 169 | 2 | 0 | 0.5611 | 0.5075 | 0.6203 |
| factor(X23.CS)=1 | 0.77 | 167 | 2 | 0 | 0.5543 | 0.5007 | 0.6137 |
| factor(X23.CS)=1 | 0.78 | 165 | 3 | 1 | 0.5443 | 0.4906 | 0.6038 |
| factor(X23.CS)=1 | 0.79 | 161 | 2 | 0 | 0.5375 | 0.4838 | 0.5972 |
| factor(X23.CS)=1 | 0.81 | 159 | 3 | 0 | 0.5274 | 0.4736 | 0.5872 |
| factor(X23.CS)=1 | 0.82 | 156 | 3 | 0 | 0.5172 | 0.4635 | 0.5772 |
| factor(X23.CS)=1 | 0.84 | 153 | 4 | 0 | 0.5037 | 0.4500 | 0.5638 |
| factor(X23.CS)=1 | 0.85 | 149 | 1 | 0 | 0.5003 | 0.4466 | 0.5605 |
| factor(X23.CS)=1 | 0.86 | 148 | 1 | 1 | 0.4969 | 0.4433 | 0.5571 |
| factor(X23.CS)=1 | 0.87 | 146 | 2 | 0 | 0.4901 | 0.4365 | 0.5503 |
| factor(X23.CS)=1 | 0.88 | 144 | 1 | 0 | 0.4867 | 0.4331 | 0.5470 |
| factor(X23.CS)=1 | 0.9 | 143 | 1 | 0 | 0.4833 | 0.4298 | 0.5436 |
| factor(X23.CS)=1 | 0.91 | 142 | 4 | 0 | 0.4697 | 0.4163 | 0.5300 |
| factor(X23.CS)=1 | 0.92 | 138 | 1 | 0 | 0.4663 | 0.4129 | 0.5266 |
| factor(X23.CS)=1 | 0.93 | 137 | 1 | 0 | 0.4629 | 0.4096 | 0.5232 |
| factor(X23.CS)=1 | 0.95 | 136 | 1 | 0 | 0.4595 | 0.4062 | 0.5198 |
| factor(X23.CS)=1 | 0.97 | 135 | 2 | 0 | 0.4527 | 0.3995 | 0.5130 |
| factor(X23.CS)=1 | 0.98 | 133 | 1 | 0 | 0.4493 | 0.3961 | 0.5096 |
| factor(X23.CS)=1 | 0.99 | 132 | 2 | 0 | 0.4425 | 0.3895 | 0.5027 |
| factor(X23.CS)=1 | 1 | 130 | 1 | 0 | 0.4391 | 0.3861 | 0.4993 |
| factor(X23.CS)=1 | 1.02 | 129 | 1 | 0 | 0.4357 | 0.3828 | 0.4959 |
| factor(X23.CS)=1 | 1.06 | 128 | 1 | 0 | 0.4323 | 0.3794 | 0.4925 |
| factor(X23.CS)=1 | 1.07 | 127 | 1 | 0 | 0.4289 | 0.3761 | 0.4890 |
| factor(X23.CS)=1 | 1.08 | 126 | 3 | 0 | 0.4187 | 0.3661 | 0.4787 |
| factor(X23.CS)=1 | 1.09 | 123 | 1 | 0 | 0.4153 | 0.3628 | 0.4753 |
| factor(X23.CS)=1 | 1.11 | 122 | 1 | 0 | 0.4118 | 0.3595 | 0.4718 |
| factor(X23.CS)=1 | 1.12 | 121 | 1 | 0 | 0.4084 | 0.3562 | 0.4684 |
| factor(X23.CS)=1 | 1.15 | 120 | 2 | 0 | 0.4016 | 0.3495 | 0.4615 |
| factor(X23.CS)=1 | 1.16 | 118 | 1 | 0 | 0.3982 | 0.3462 | 0.4580 |
| factor(X23.CS)=1 | 1.17 | 117 | 2 | 0 | 0.3914 | 0.3396 | 0.4511 |
| factor(X23.CS)=1 | 1.18 | 115 | 1 | 0 | 0.3880 | 0.3363 | 0.4477 |
| factor(X23.CS)=1 | 1.2 | 114 | 2 | 0 | 0.3812 | 0.3297 | 0.4407 |
| factor(X23.CS)=1 | 1.25 | 112 | 2 | 0 | 0.3744 | 0.3232 | 0.4338 |
| factor(X23.CS)=1 | 1.3 | 110 | 1 | 0 | 0.3710 | 0.3199 | 0.4303 |
| factor(X23.CS)=1 | 1.31 | 109 | 2 | 0 | 0.3642 | 0.3133 | 0.4233 |
| factor(X23.CS)=1 | 1.35 | 107 | 2 | 0 | 0.3574 | 0.3068 | 0.4164 |
| factor(X23.CS)=1 | 1.36 | 105 | 1 | 0 | 0.3540 | 0.3035 | 0.4129 |
| factor(X23.CS)=1 | 1.38 | 104 | 2 | 0 | 0.3472 | 0.2970 | 0.4059 |
| factor(X23.CS)=1 | 1.39 | 102 | 2 | 1 | 0.3404 | 0.2904 | 0.3989 |
| factor(X23.CS)=1 | 1.42 | 99 | 1 | 0 | 0.3369 | 0.2872 | 0.3953 |
| factor(X23.CS)=1 | 1.45 | 98 | 1 | 0 | 0.3335 | 0.2839 | 0.3918 |
| factor(X23.CS)=1 | 1.46 | 97 | 1 | 0 | 0.3301 | 0.2806 | 0.3883 |
| factor(X23.CS)=1 | 1.5 | 96 | 2 | 0 | 0.3232 | 0.2740 | 0.3812 |
| factor(X23.CS)=1 | 1.55 | 94 | 2 | 0 | 0.3163 | 0.2675 | 0.3740 |
| factor(X23.CS)=1 | 1.58 | 92 | 1 | 0 | 0.3129 | 0.2642 | 0.3705 |
| factor(X23.CS)=1 | 1.72 | 91 | 1 | 0 | 0.3094 | 0.2609 | 0.3669 |
| factor(X23.CS)=1 | 1.74 | 90 | 1 | 0 | 0.3060 | 0.2577 | 0.3634 |
| factor(X23.CS)=1 | 1.79 | 89 | 1 | 0 | 0.3026 | 0.2544 | 0.3598 |
| factor(X23.CS)=1 | 1.92 | 88 | 1 | 0 | 0.2991 | 0.2512 | 0.3562 |
| factor(X23.CS)=1 | 1.98 | 87 | 4 | 0 | 0.2854 | 0.2382 | 0.3419 |
| factor(X23.CS)=1 | 2.09 | 83 | 1 | 0 | 0.2819 | 0.2350 | 0.3383 |
| factor(X23.CS)=1 | 2.26 | 82 | 1 | 0 | 0.2785 | 0.2317 | 0.3347 |
| factor(X23.CS)=1 | 2.32 | 81 | 0 | 1 | 0.2785 | 0.2317 | 0.3347 |
| factor(X23.CS)=1 | 2.53 | 80 | 1 | 0 | 0.2750 | 0.2285 | 0.3310 |
| factor(X23.CS)=1 | 2.71 | 79 | 0 | 1 | 0.2750 | 0.2285 | 0.3310 |
| factor(X23.CS)=1 | 2.72 | 78 | 3 | 0 | 0.2644 | 0.2185 | 0.3200 |
| factor(X23.CS)=1 | 2.83 | 75 | 1 | 0 | 0.2609 | 0.2152 | 0.3163 |
| factor(X23.CS)=1 | 2.95 | 74 | 0 | 1 | 0.2609 | 0.2152 | 0.3163 |
| factor(X23.CS)=1 | 2.99 | 73 | 1 | 0 | 0.2573 | 0.2119 | 0.3125 |
| factor(X23.CS)=1 | 3 | 72 | 0 | 1 | 0.2573 | 0.2119 | 0.3125 |
| factor(X23.CS)=1 | 3.05 | 71 | 0 | 1 | 0.2573 | 0.2119 | 0.3125 |
| factor(X23.CS)=1 | 3.18 | 70 | 0 | 1 | 0.2573 | 0.2119 | 0.3125 |
| factor(X23.CS)=1 | 3.25 | 69 | 0 | 1 | 0.2573 | 0.2119 | 0.3125 |
| factor(X23.CS)=1 | 3.39 | 68 | 2 | 1 | 0.2498 | 0.2047 | 0.3047 |
| factor(X23.CS)=1 | 3.53 | 65 | 0 | 1 | 0.2498 | 0.2047 | 0.3047 |
| factor(X23.CS)=1 | 3.68 | 64 | 0 | 1 | 0.2498 | 0.2047 | 0.3047 |
| factor(X23.CS)=1 | 3.87 | 63 | 0 | 1 | 0.2498 | 0.2047 | 0.3047 |
| factor(X23.CS)=1 | 3.88 | 62 | 0 | 1 | 0.2498 | 0.2047 | 0.3047 |
| factor(X23.CS)=1 | 4.04 | 61 | 0 | 1 | 0.2498 | 0.2047 | 0.3047 |
| factor(X23.CS)=1 | 4.16 | 60 | 0 | 1 | 0.2498 | 0.2047 | 0.3047 |
| factor(X23.CS)=1 | 4.22 | 59 | 0 | 1 | 0.2498 | 0.2047 | 0.3047 |
| factor(X23.CS)=1 | 4.33 | 58 | 0 | 1 | 0.2498 | 0.2047 | 0.3047 |
| factor(X23.CS)=1 | 4.41 | 57 | 0 | 1 | 0.2498 | 0.2047 | 0.3047 |
| factor(X23.CS)=1 | 4.44 | 56 | 0 | 1 | 0.2498 | 0.2047 | 0.3047 |
| factor(X23.CS)=1 | 4.47 | 55 | 0 | 1 | 0.2498 | 0.2047 | 0.3047 |
| factor(X23.CS)=1 | 4.48 | 54 | 0 | 1 | 0.2498 | 0.2047 | 0.3047 |
| factor(X23.CS)=1 | 4.53 | 53 | 0 | 2 | 0.2498 | 0.2047 | 0.3047 |
| factor(X23.CS)=1 | 4.55 | 51 | 1 | 1 | 0.2449 | 0.2000 | 0.2998 |
| factor(X23.CS)=1 | 4.6 | 49 | 0 | 1 | 0.2449 | 0.2000 | 0.2998 |
| factor(X23.CS)=1 | 4.61 | 48 | 0 | 1 | 0.2449 | 0.2000 | 0.2998 |
| factor(X23.CS)=1 | 4.65 | 47 | 0 | 1 | 0.2449 | 0.2000 | 0.2998 |
| factor(X23.CS)=1 | 4.68 | 46 | 1 | 0 | 0.2395 | 0.1947 | 0.2946 |
| factor(X23.CS)=1 | 4.7 | 45 | 0 | 1 | 0.2395 | 0.1947 | 0.2946 |
| factor(X23.CS)=1 | 4.72 | 44 | 1 | 0 | 0.2341 | 0.1894 | 0.2893 |
| factor(X23.CS)=1 | 4.81 | 43 | 0 | 1 | 0.2341 | 0.1894 | 0.2893 |
| factor(X23.CS)=1 | 4.88 | 42 | 0 | 1 | 0.2341 | 0.1894 | 0.2893 |
| factor(X23.CS)=1 | 4.89 | 41 | 0 | 1 | 0.2341 | 0.1894 | 0.2893 |
| factor(X23.CS)=1 | 5 | 40 | 0 | 40 | 0.2341 | 0.1894 | 0.2893 |

|  |  |  |  |  |  |  |  |  |  |
| --- | --- | --- | --- | --- | --- | --- | --- | --- | --- |
| X23.CS | records | n.max | n.start | events | \*rmean | \*se(rmean) | median | 0.95LCL | 0.95UCL |
| factor(X23.CS)=0 | 906 | 906 | 906 | 378 | 3.318 | 0.067 | NA | NA | NA |
| factor(X23.CS)=1 | 299 | 299 | 299 | 225 | 1.82 | 0.111 | 0.86 | 0.78 | 1 |

Landmark analysis for time segment: EFS(years) > 5
Log rank test: implements the G-rho family of Harrington and Fleming (1982), with weights on each death of S(t)^rho, where S is the Kaplan-Meier estimate of survival. With rho = 0 this is the log-rank or Mantel-Haenszel test.

|  |  |  |  |
| --- | --- | --- | --- |
|  | N | Observed | Expected |
| factor(X23.CS)=0 | 297 | 5 | 6.9635 |
| factor(X23.CS)=1 | 40 | 3 | 1.0365 |

Chisq=
4.2986
on
1
degree of freedom, p=
0.0381
Survival table

|  |  |  |  |  |  |  |  |
| --- | --- | --- | --- | --- | --- | --- | --- |
|  | EFS(years) | N.Risk | N.Event | N.Censor | Survival | 95%CI Low | 95%CI Upp |
| factor(X23.CS)=0 | 5.02 | 297 | 0 | 1 | 1.0000 | 1.0000 | 1.0000 |
| factor(X23.CS)=0 | 5.03 | 296 | 0 | 1 | 1.0000 | 1.0000 | 1.0000 |
| factor(X23.CS)=0 | 5.04 | 295 | 0 | 1 | 1.0000 | 1.0000 | 1.0000 |
| factor(X23.CS)=0 | 5.05 | 294 | 0 | 2 | 1.0000 | 1.0000 | 1.0000 |
| factor(X23.CS)=0 | 5.08 | 292 | 0 | 1 | 1.0000 | 1.0000 | 1.0000 |
| factor(X23.CS)=0 | 5.1 | 291 | 0 | 1 | 1.0000 | 1.0000 | 1.0000 |
| factor(X23.CS)=0 | 5.12 | 290 | 0 | 1 | 1.0000 | 1.0000 | 1.0000 |
| factor(X23.CS)=0 | 5.14 | 289 | 0 | 1 | 1.0000 | 1.0000 | 1.0000 |
| factor(X23.CS)=0 | 5.15 | 288 | 0 | 2 | 1.0000 | 1.0000 | 1.0000 |
| factor(X23.CS)=0 | 5.16 | 286 | 0 | 1 | 1.0000 | 1.0000 | 1.0000 |
| factor(X23.CS)=0 | 5.17 | 285 | 0 | 1 | 1.0000 | 1.0000 | 1.0000 |
| factor(X23.CS)=0 | 5.18 | 284 | 0 | 3 | 1.0000 | 1.0000 | 1.0000 |
| factor(X23.CS)=0 | 5.2 | 281 | 0 | 1 | 1.0000 | 1.0000 | 1.0000 |
| factor(X23.CS)=0 | 5.22 | 280 | 0 | 1 | 1.0000 | 1.0000 | 1.0000 |
| factor(X23.CS)=0 | 5.25 | 279 | 0 | 2 | 1.0000 | 1.0000 | 1.0000 |
| factor(X23.CS)=0 | 5.26 | 277 | 0 | 1 | 1.0000 | 1.0000 | 1.0000 |
| factor(X23.CS)=0 | 5.27 | 276 | 0 | 2 | 1.0000 | 1.0000 | 1.0000 |
| factor(X23.CS)=0 | 5.29 | 274 | 0 | 2 | 1.0000 | 1.0000 | 1.0000 |
| factor(X23.CS)=0 | 5.3 | 272 | 0 | 1 | 1.0000 | 1.0000 | 1.0000 |
| factor(X23.CS)=0 | 5.31 | 271 | 0 | 1 | 1.0000 | 1.0000 | 1.0000 |
| factor(X23.CS)=0 | 5.32 | 270 | 0 | 2 | 1.0000 | 1.0000 | 1.0000 |
| factor(X23.CS)=0 | 5.33 | 268 | 0 | 2 | 1.0000 | 1.0000 | 1.0000 |
| factor(X23.CS)=0 | 5.34 | 266 | 0 | 1 | 1.0000 | 1.0000 | 1.0000 |
| factor(X23.CS)=0 | 5.35 | 265 | 0 | 6 | 1.0000 | 1.0000 | 1.0000 |
| factor(X23.CS)=0 | 5.36 | 259 | 0 | 1 | 1.0000 | 1.0000 | 1.0000 |
| factor(X23.CS)=0 | 5.37 | 258 | 0 | 2 | 1.0000 | 1.0000 | 1.0000 |
| factor(X23.CS)=0 | 5.38 | 256 | 0 | 2 | 1.0000 | 1.0000 | 1.0000 |
| factor(X23.CS)=0 | 5.39 | 254 | 0 | 1 | 1.0000 | 1.0000 | 1.0000 |
| factor(X23.CS)=0 | 5.4 | 253 | 0 | 1 | 1.0000 | 1.0000 | 1.0000 |
| factor(X23.CS)=0 | 5.41 | 252 | 0 | 2 | 1.0000 | 1.0000 | 1.0000 |
| factor(X23.CS)=0 | 5.42 | 250 | 0 | 3 | 1.0000 | 1.0000 | 1.0000 |
| factor(X23.CS)=0 | 5.43 | 247 | 0 | 1 | 1.0000 | 1.0000 | 1.0000 |
| factor(X23.CS)=0 | 5.44 | 246 | 0 | 4 | 1.0000 | 1.0000 | 1.0000 |
| factor(X23.CS)=0 | 5.45 | 242 | 0 | 1 | 1.0000 | 1.0000 | 1.0000 |
| factor(X23.CS)=0 | 5.46 | 241 | 0 | 2 | 1.0000 | 1.0000 | 1.0000 |
| factor(X23.CS)=0 | 5.48 | 239 | 0 | 2 | 1.0000 | 1.0000 | 1.0000 |
| factor(X23.CS)=0 | 5.49 | 237 | 0 | 1 | 1.0000 | 1.0000 | 1.0000 |
| factor(X23.CS)=0 | 5.5 | 236 | 0 | 1 | 1.0000 | 1.0000 | 1.0000 |
| factor(X23.CS)=0 | 5.51 | 235 | 0 | 2 | 1.0000 | 1.0000 | 1.0000 |
| factor(X23.CS)=0 | 5.52 | 233 | 0 | 2 | 1.0000 | 1.0000 | 1.0000 |
| factor(X23.CS)=0 | 5.54 | 231 | 0 | 4 | 1.0000 | 1.0000 | 1.0000 |
| factor(X23.CS)=0 | 5.55 | 227 | 0 | 1 | 1.0000 | 1.0000 | 1.0000 |
| factor(X23.CS)=0 | 5.56 | 226 | 0 | 2 | 1.0000 | 1.0000 | 1.0000 |
| factor(X23.CS)=0 | 5.57 | 224 | 0 | 1 | 1.0000 | 1.0000 | 1.0000 |
| factor(X23.CS)=0 | 5.58 | 223 | 1 | 1 | 0.9955 | 0.9868 | 1.0000 |
| factor(X23.CS)=0 | 5.6 | 221 | 0 | 1 | 0.9955 | 0.9868 | 1.0000 |
| factor(X23.CS)=0 | 5.61 | 220 | 0 | 1 | 0.9955 | 0.9868 | 1.0000 |
| factor(X23.CS)=0 | 5.63 | 219 | 0 | 2 | 0.9955 | 0.9868 | 1.0000 |
| factor(X23.CS)=0 | 5.65 | 217 | 0 | 1 | 0.9955 | 0.9868 | 1.0000 |
| factor(X23.CS)=0 | 5.66 | 216 | 0 | 1 | 0.9955 | 0.9868 | 1.0000 |
| factor(X23.CS)=0 | 5.67 | 215 | 0 | 3 | 0.9955 | 0.9868 | 1.0000 |
| factor(X23.CS)=0 | 5.7 | 212 | 0 | 3 | 0.9955 | 0.9868 | 1.0000 |
| factor(X23.CS)=0 | 5.71 | 209 | 0 | 2 | 0.9955 | 0.9868 | 1.0000 |
| factor(X23.CS)=0 | 5.72 | 207 | 0 | 1 | 0.9955 | 0.9868 | 1.0000 |
| factor(X23.CS)=0 | 5.73 | 206 | 0 | 2 | 0.9955 | 0.9868 | 1.0000 |
| factor(X23.CS)=0 | 5.74 | 204 | 0 | 1 | 0.9955 | 0.9868 | 1.0000 |
| factor(X23.CS)=0 | 5.75 | 203 | 0 | 2 | 0.9955 | 0.9868 | 1.0000 |
| factor(X23.CS)=0 | 5.77 | 201 | 0 | 1 | 0.9955 | 0.9868 | 1.0000 |
| factor(X23.CS)=0 | 5.78 | 200 | 0 | 2 | 0.9955 | 0.9868 | 1.0000 |
| factor(X23.CS)=0 | 5.79 | 198 | 0 | 1 | 0.9955 | 0.9868 | 1.0000 |
| factor(X23.CS)=0 | 5.8 | 197 | 0 | 1 | 0.9955 | 0.9868 | 1.0000 |
| factor(X23.CS)=0 | 5.81 | 196 | 0 | 2 | 0.9955 | 0.9868 | 1.0000 |
| factor(X23.CS)=0 | 5.84 | 194 | 0 | 2 | 0.9955 | 0.9868 | 1.0000 |
| factor(X23.CS)=0 | 5.85 | 192 | 0 | 1 | 0.9955 | 0.9868 | 1.0000 |
| factor(X23.CS)=0 | 5.88 | 191 | 0 | 1 | 0.9955 | 0.9868 | 1.0000 |
| factor(X23.CS)=0 | 5.89 | 190 | 0 | 1 | 0.9955 | 0.9868 | 1.0000 |
| factor(X23.CS)=0 | 5.92 | 189 | 0 | 1 | 0.9955 | 0.9868 | 1.0000 |
| factor(X23.CS)=0 | 5.93 | 188 | 0 | 1 | 0.9955 | 0.9868 | 1.0000 |
| factor(X23.CS)=0 | 5.94 | 187 | 0 | 2 | 0.9955 | 0.9868 | 1.0000 |
| factor(X23.CS)=0 | 5.95 | 185 | 0 | 3 | 0.9955 | 0.9868 | 1.0000 |
| factor(X23.CS)=0 | 5.96 | 182 | 0 | 1 | 0.9955 | 0.9868 | 1.0000 |
| factor(X23.CS)=0 | 5.98 | 181 | 0 | 1 | 0.9955 | 0.9868 | 1.0000 |
| factor(X23.CS)=0 | 6 | 180 | 0 | 6 | 0.9955 | 0.9868 | 1.0000 |
| factor(X23.CS)=0 | 6.01 | 174 | 0 | 1 | 0.9955 | 0.9868 | 1.0000 |
| factor(X23.CS)=0 | 6.02 | 173 | 0 | 1 | 0.9955 | 0.9868 | 1.0000 |
| factor(X23.CS)=0 | 6.04 | 172 | 0 | 1 | 0.9955 | 0.9868 | 1.0000 |
| factor(X23.CS)=0 | 6.07 | 171 | 0 | 1 | 0.9955 | 0.9868 | 1.0000 |
| factor(X23.CS)=0 | 6.08 | 170 | 0 | 1 | 0.9955 | 0.9868 | 1.0000 |
| factor(X23.CS)=0 | 6.12 | 169 | 0 | 2 | 0.9955 | 0.9868 | 1.0000 |
| factor(X23.CS)=0 | 6.15 | 167 | 0 | 3 | 0.9955 | 0.9868 | 1.0000 |
| factor(X23.CS)=0 | 6.16 | 164 | 0 | 1 | 0.9955 | 0.9868 | 1.0000 |
| factor(X23.CS)=0 | 6.17 | 163 | 0 | 1 | 0.9955 | 0.9868 | 1.0000 |
| factor(X23.CS)=0 | 6.18 | 162 | 0 | 2 | 0.9955 | 0.9868 | 1.0000 |
| factor(X23.CS)=0 | 6.22 | 160 | 0 | 1 | 0.9955 | 0.9868 | 1.0000 |
| factor(X23.CS)=0 | 6.25 | 159 | 0 | 2 | 0.9955 | 0.9868 | 1.0000 |
| factor(X23.CS)=0 | 6.27 | 157 | 0 | 1 | 0.9955 | 0.9868 | 1.0000 |
| factor(X23.CS)=0 | 6.28 | 156 | 0 | 1 | 0.9955 | 0.9868 | 1.0000 |
| factor(X23.CS)=0 | 6.29 | 155 | 0 | 1 | 0.9955 | 0.9868 | 1.0000 |
| factor(X23.CS)=0 | 6.32 | 154 | 0 | 1 | 0.9955 | 0.9868 | 1.0000 |
| factor(X23.CS)=0 | 6.35 | 153 | 0 | 2 | 0.9955 | 0.9868 | 1.0000 |
| factor(X23.CS)=0 | 6.36 | 151 | 0 | 1 | 0.9955 | 0.9868 | 1.0000 |
| factor(X23.CS)=0 | 6.37 | 150 | 0 | 2 | 0.9955 | 0.9868 | 1.0000 |
| factor(X23.CS)=0 | 6.38 | 148 | 0 | 1 | 0.9955 | 0.9868 | 1.0000 |
| factor(X23.CS)=0 | 6.39 | 147 | 0 | 3 | 0.9955 | 0.9868 | 1.0000 |
| factor(X23.CS)=0 | 6.4 | 144 | 0 | 2 | 0.9955 | 0.9868 | 1.0000 |
| factor(X23.CS)=0 | 6.42 | 142 | 0 | 3 | 0.9955 | 0.9868 | 1.0000 |
| factor(X23.CS)=0 | 6.45 | 139 | 3 | 1 | 0.9740 | 0.9488 | 0.9999 |
| factor(X23.CS)=0 | 6.46 | 135 | 0 | 2 | 0.9740 | 0.9488 | 0.9999 |
| factor(X23.CS)=0 | 6.47 | 133 | 0 | 3 | 0.9740 | 0.9488 | 0.9999 |
| factor(X23.CS)=0 | 6.48 | 130 | 0 | 2 | 0.9740 | 0.9488 | 0.9999 |
| factor(X23.CS)=0 | 6.49 | 128 | 0 | 1 | 0.9740 | 0.9488 | 0.9999 |
| factor(X23.CS)=0 | 6.5 | 127 | 0 | 2 | 0.9740 | 0.9488 | 0.9999 |
| factor(X23.CS)=0 | 6.51 | 125 | 0 | 2 | 0.9740 | 0.9488 | 0.9999 |
| factor(X23.CS)=0 | 6.52 | 123 | 0 | 1 | 0.9740 | 0.9488 | 0.9999 |
| factor(X23.CS)=0 | 6.53 | 122 | 0 | 1 | 0.9740 | 0.9488 | 0.9999 |
| factor(X23.CS)=0 | 6.54 | 121 | 0 | 1 | 0.9740 | 0.9488 | 0.9999 |
| factor(X23.CS)=0 | 6.55 | 120 | 0 | 1 | 0.9740 | 0.9488 | 0.9999 |
| factor(X23.CS)=0 | 6.57 | 119 | 0 | 2 | 0.9740 | 0.9488 | 0.9999 |
| factor(X23.CS)=0 | 6.58 | 117 | 0 | 2 | 0.9740 | 0.9488 | 0.9999 |
| factor(X23.CS)=0 | 6.59 | 115 | 0 | 1 | 0.9740 | 0.9488 | 0.9999 |
| factor(X23.CS)=0 | 6.6 | 114 | 0 | 1 | 0.9740 | 0.9488 | 0.9999 |
| factor(X23.CS)=0 | 6.63 | 113 | 0 | 1 | 0.9740 | 0.9488 | 0.9999 |
| factor(X23.CS)=0 | 6.64 | 112 | 0 | 2 | 0.9740 | 0.9488 | 0.9999 |
| factor(X23.CS)=0 | 6.65 | 110 | 0 | 3 | 0.9740 | 0.9488 | 0.9999 |
| factor(X23.CS)=0 | 6.68 | 107 | 0 | 1 | 0.9740 | 0.9488 | 0.9999 |
| factor(X23.CS)=0 | 6.69 | 106 | 0 | 1 | 0.9740 | 0.9488 | 0.9999 |
| factor(X23.CS)=0 | 6.73 | 105 | 0 | 1 | 0.9740 | 0.9488 | 0.9999 |
| factor(X23.CS)=0 | 6.75 | 104 | 0 | 3 | 0.9740 | 0.9488 | 0.9999 |
| factor(X23.CS)=0 | 6.77 | 101 | 0 | 1 | 0.9740 | 0.9488 | 0.9999 |
| factor(X23.CS)=0 | 6.78 | 100 | 0 | 1 | 0.9740 | 0.9488 | 0.9999 |
| factor(X23.CS)=0 | 6.79 | 99 | 0 | 1 | 0.9740 | 0.9488 | 0.9999 |
| factor(X23.CS)=0 | 6.81 | 98 | 0 | 2 | 0.9740 | 0.9488 | 0.9999 |
| factor(X23.CS)=0 | 6.85 | 96 | 0 | 1 | 0.9740 | 0.9488 | 0.9999 |
| factor(X23.CS)=0 | 6.89 | 95 | 0 | 2 | 0.9740 | 0.9488 | 0.9999 |
| factor(X23.CS)=0 | 6.91 | 93 | 0 | 1 | 0.9740 | 0.9488 | 0.9999 |
| factor(X23.CS)=0 | 6.92 | 92 | 0 | 1 | 0.9740 | 0.9488 | 0.9999 |
| factor(X23.CS)=0 | 6.94 | 91 | 0 | 1 | 0.9740 | 0.9488 | 0.9999 |
| factor(X23.CS)=0 | 6.96 | 90 | 0 | 4 | 0.9740 | 0.9488 | 0.9999 |
| factor(X23.CS)=0 | 6.98 | 86 | 0 | 1 | 0.9740 | 0.9488 | 0.9999 |
| factor(X23.CS)=0 | 7 | 85 | 0 | 3 | 0.9740 | 0.9488 | 0.9999 |
| factor(X23.CS)=0 | 7.08 | 82 | 0 | 1 | 0.9740 | 0.9488 | 0.9999 |
| factor(X23.CS)=0 | 7.14 | 81 | 0 | 1 | 0.9740 | 0.9488 | 0.9999 |
| factor(X23.CS)=0 | 7.16 | 80 | 0 | 1 | 0.9740 | 0.9488 | 0.9999 |
| factor(X23.CS)=0 | 7.2 | 79 | 0 | 1 | 0.9740 | 0.9488 | 0.9999 |
| factor(X23.CS)=0 | 7.21 | 78 | 0 | 1 | 0.9740 | 0.9488 | 0.9999 |
| factor(X23.CS)=0 | 7.26 | 77 | 0 | 3 | 0.9740 | 0.9488 | 0.9999 |
| factor(X23.CS)=0 | 7.29 | 74 | 0 | 1 | 0.9740 | 0.9488 | 0.9999 |
| factor(X23.CS)=0 | 7.35 | 73 | 0 | 2 | 0.9740 | 0.9488 | 0.9999 |
| factor(X23.CS)=0 | 7.36 | 71 | 0 | 4 | 0.9740 | 0.9488 | 0.9999 |
| factor(X23.CS)=0 | 7.37 | 67 | 0 | 1 | 0.9740 | 0.9488 | 0.9999 |
| factor(X23.CS)=0 | 7.38 | 66 | 0 | 1 | 0.9740 | 0.9488 | 0.9999 |
| factor(X23.CS)=0 | 7.39 | 65 | 0 | 2 | 0.9740 | 0.9488 | 0.9999 |
| factor(X23.CS)=0 | 7.4 | 63 | 0 | 1 | 0.9740 | 0.9488 | 0.9999 |
| factor(X23.CS)=0 | 7.41 | 62 | 0 | 1 | 0.9740 | 0.9488 | 0.9999 |
| factor(X23.CS)=0 | 7.42 | 61 | 0 | 1 | 0.9740 | 0.9488 | 0.9999 |
| factor(X23.CS)=0 | 7.45 | 60 | 0 | 1 | 0.9740 | 0.9488 | 0.9999 |
| factor(X23.CS)=0 | 7.47 | 59 | 0 | 1 | 0.9740 | 0.9488 | 0.9999 |
| factor(X23.CS)=0 | 7.48 | 58 | 0 | 2 | 0.9740 | 0.9488 | 0.9999 |
| factor(X23.CS)=0 | 7.5 | 56 | 0 | 1 | 0.9740 | 0.9488 | 0.9999 |
| factor(X23.CS)=0 | 7.53 | 55 | 0 | 1 | 0.9740 | 0.9488 | 0.9999 |
| factor(X23.CS)=0 | 7.54 | 54 | 0 | 1 | 0.9740 | 0.9488 | 0.9999 |
| factor(X23.CS)=0 | 7.55 | 53 | 0 | 1 | 0.9740 | 0.9488 | 0.9999 |
| factor(X23.CS)=0 | 7.56 | 52 | 0 | 2 | 0.9740 | 0.9488 | 0.9999 |
| factor(X23.CS)=0 | 7.58 | 50 | 0 | 4 | 0.9740 | 0.9488 | 0.9999 |
| factor(X23.CS)=0 | 7.59 | 46 | 0 | 1 | 0.9740 | 0.9488 | 0.9999 |
| factor(X23.CS)=0 | 7.6 | 45 | 0 | 2 | 0.9740 | 0.9488 | 0.9999 |
| factor(X23.CS)=0 | 7.61 | 43 | 0 | 1 | 0.9740 | 0.9488 | 0.9999 |
| factor(X23.CS)=0 | 7.62 | 42 | 0 | 1 | 0.9740 | 0.9488 | 0.9999 |
| factor(X23.CS)=0 | 7.67 | 41 | 0 | 1 | 0.9740 | 0.9488 | 0.9999 |
| factor(X23.CS)=0 | 7.68 | 40 | 0 | 1 | 0.9740 | 0.9488 | 0.9999 |
| factor(X23.CS)=0 | 7.69 | 39 | 0 | 2 | 0.9740 | 0.9488 | 0.9999 |
| factor(X23.CS)=0 | 7.73 | 37 | 0 | 2 | 0.9740 | 0.9488 | 0.9999 |
| factor(X23.CS)=0 | 7.74 | 35 | 0 | 3 | 0.9740 | 0.9488 | 0.9999 |
| factor(X23.CS)=0 | 7.81 | 32 | 0 | 1 | 0.9740 | 0.9488 | 0.9999 |
| factor(X23.CS)=0 | 7.82 | 31 | 0 | 1 | 0.9740 | 0.9488 | 0.9999 |
| factor(X23.CS)=0 | 7.94 | 30 | 0 | 2 | 0.9740 | 0.9488 | 0.9999 |
| factor(X23.CS)=0 | 7.98 | 28 | 0 | 1 | 0.9740 | 0.9488 | 0.9999 |
| factor(X23.CS)=0 | 7.99 | 27 | 1 | 1 | 0.9380 | 0.8672 | 1.0000 |
| factor(X23.CS)=0 | 8.01 | 25 | 0 | 1 | 0.9380 | 0.8672 | 1.0000 |
| factor(X23.CS)=0 | 8.07 | 24 | 0 | 1 | 0.9380 | 0.8672 | 1.0000 |
| factor(X23.CS)=0 | 8.15 | 23 | 0 | 1 | 0.9380 | 0.8672 | 1.0000 |
| factor(X23.CS)=0 | 8.17 | 22 | 0 | 1 | 0.9380 | 0.8672 | 1.0000 |
| factor(X23.CS)=0 | 8.21 | 21 | 0 | 1 | 0.9380 | 0.8672 | 1.0000 |
| factor(X23.CS)=0 | 8.26 | 20 | 0 | 1 | 0.9380 | 0.8672 | 1.0000 |
| factor(X23.CS)=0 | 8.27 | 19 | 0 | 1 | 0.9380 | 0.8672 | 1.0000 |
| factor(X23.CS)=0 | 8.28 | 18 | 0 | 1 | 0.9380 | 0.8672 | 1.0000 |
| factor(X23.CS)=0 | 8.29 | 17 | 0 | 1 | 0.9380 | 0.8672 | 1.0000 |
| factor(X23.CS)=0 | 8.48 | 16 | 0 | 1 | 0.9380 | 0.8672 | 1.0000 |
| factor(X23.CS)=0 | 8.49 | 15 | 0 | 2 | 0.9380 | 0.8672 | 1.0000 |
| factor(X23.CS)=0 | 8.5 | 13 | 0 | 1 | 0.9380 | 0.8672 | 1.0000 |
| factor(X23.CS)=0 | 8.53 | 12 | 0 | 1 | 0.9380 | 0.8672 | 1.0000 |
| factor(X23.CS)=0 | 8.72 | 11 | 0 | 1 | 0.9380 | 0.8672 | 1.0000 |
| factor(X23.CS)=0 | 8.84 | 10 | 0 | 1 | 0.9380 | 0.8672 | 1.0000 |
| factor(X23.CS)=0 | 8.88 | 9 | 0 | 2 | 0.9380 | 0.8672 | 1.0000 |
| factor(X23.CS)=0 | 9.04 | 7 | 0 | 1 | 0.9380 | 0.8672 | 1.0000 |
| factor(X23.CS)=0 | 9.06 | 6 | 0 | 2 | 0.9380 | 0.8672 | 1.0000 |
| factor(X23.CS)=0 | 9.08 | 4 | 0 | 1 | 0.9380 | 0.8672 | 1.0000 |
| factor(X23.CS)=0 | 9.26 | 3 | 0 | 1 | 0.9380 | 0.8672 | 1.0000 |
| factor(X23.CS)=0 | 9.61 | 2 | 0 | 1 | 0.9380 | 0.8672 | 1.0000 |
| factor(X23.CS)=0 | 9.95 | 1 | 0 | 1 | 0.9380 | 0.8672 | 1.0000 |
| factor(X23.CS)=1 | 5.12 | 40 | 1 | 0 | 0.9750 | 0.9278 | 1.0000 |
| factor(X23.CS)=1 | 5.2 | 39 | 0 | 1 | 0.9750 | 0.9278 | 1.0000 |
| factor(X23.CS)=1 | 5.26 | 38 | 0 | 1 | 0.9750 | 0.9278 | 1.0000 |
| factor(X23.CS)=1 | 5.33 | 37 | 0 | 1 | 0.9750 | 0.9278 | 1.0000 |
| factor(X23.CS)=1 | 5.34 | 36 | 0 | 1 | 0.9750 | 0.9278 | 1.0000 |
| factor(X23.CS)=1 | 5.36 | 35 | 0 | 1 | 0.9750 | 0.9278 | 1.0000 |
| factor(X23.CS)=1 | 5.38 | 34 | 0 | 1 | 0.9750 | 0.9278 | 1.0000 |
| factor(X23.CS)=1 | 5.4 | 33 | 0 | 1 | 0.9750 | 0.9278 | 1.0000 |
| factor(X23.CS)=1 | 5.42 | 32 | 0 | 1 | 0.9750 | 0.9278 | 1.0000 |
| factor(X23.CS)=1 | 5.48 | 31 | 0 | 1 | 0.9750 | 0.9278 | 1.0000 |
| factor(X23.CS)=1 | 5.55 | 30 | 0 | 1 | 0.9750 | 0.9278 | 1.0000 |
| factor(X23.CS)=1 | 5.81 | 29 | 1 | 0 | 0.9414 | 0.8648 | 1.0000 |
| factor(X23.CS)=1 | 5.89 | 28 | 0 | 1 | 0.9414 | 0.8648 | 1.0000 |
| factor(X23.CS)=1 | 6.04 | 27 | 0 | 1 | 0.9414 | 0.8648 | 1.0000 |
| factor(X23.CS)=1 | 6.16 | 26 | 0 | 1 | 0.9414 | 0.8648 | 1.0000 |
| factor(X23.CS)=1 | 6.19 | 25 | 0 | 1 | 0.9414 | 0.8648 | 1.0000 |
| factor(X23.CS)=1 | 6.2 | 24 | 0 | 1 | 0.9414 | 0.8648 | 1.0000 |
| factor(X23.CS)=1 | 6.38 | 23 | 0 | 1 | 0.9414 | 0.8648 | 1.0000 |
| factor(X23.CS)=1 | 6.39 | 22 | 0 | 1 | 0.9414 | 0.8648 | 1.0000 |
| factor(X23.CS)=1 | 6.4 | 21 | 0 | 1 | 0.9414 | 0.8648 | 1.0000 |
| factor(X23.CS)=1 | 6.51 | 20 | 0 | 1 | 0.9414 | 0.8648 | 1.0000 |
| factor(X23.CS)=1 | 6.56 | 19 | 1 | 1 | 0.8918 | 0.7786 | 1.0000 |
| factor(X23.CS)=1 | 6.79 | 17 | 0 | 1 | 0.8918 | 0.7786 | 1.0000 |
| factor(X23.CS)=1 | 6.87 | 16 | 0 | 1 | 0.8918 | 0.7786 | 1.0000 |
| factor(X23.CS)=1 | 7.12 | 15 | 0 | 1 | 0.8918 | 0.7786 | 1.0000 |
| factor(X23.CS)=1 | 7.27 | 14 | 0 | 1 | 0.8918 | 0.7786 | 1.0000 |
| factor(X23.CS)=1 | 7.36 | 13 | 0 | 2 | 0.8918 | 0.7786 | 1.0000 |
| factor(X23.CS)=1 | 7.46 | 11 | 0 | 1 | 0.8918 | 0.7786 | 1.0000 |
| factor(X23.CS)=1 | 7.48 | 10 | 0 | 1 | 0.8918 | 0.7786 | 1.0000 |
| factor(X23.CS)=1 | 7.72 | 9 | 0 | 1 | 0.8918 | 0.7786 | 1.0000 |
| factor(X23.CS)=1 | 7.74 | 8 | 0 | 2 | 0.8918 | 0.7786 | 1.0000 |
| factor(X23.CS)=1 | 7.9 | 6 | 0 | 1 | 0.8918 | 0.7786 | 1.0000 |
| factor(X23.CS)=1 | 8.06 | 5 | 0 | 1 | 0.8918 | 0.7786 | 1.0000 |
| factor(X23.CS)=1 | 8.1 | 4 | 0 | 1 | 0.8918 | 0.7786 | 1.0000 |
| factor(X23.CS)=1 | 8.36 | 3 | 0 | 1 | 0.8918 | 0.7786 | 1.0000 |
| factor(X23.CS)=1 | 8.69 | 2 | 0 | 1 | 0.8918 | 0.7786 | 1.0000 |
| factor(X23.CS)=1 | 8.88 | 1 | 0 | 1 | 0.8918 | 0.7786 | 1.0000 |

|  |  |  |  |  |  |  |  |  |  |
| --- | --- | --- | --- | --- | --- | --- | --- | --- | --- |
| X23.CS | records | n.max | n.start | events | \*rmean | \*se(rmean) | median | 0.95LCL | 0.95UCL |
| factor(X23.CS)=0 | 297 | 297 | 297 | 5 | 9.283 | 0.064 | NA | NA | NA |
| factor(X23.CS)=1 | 40 | 40 | 40 | 3 | 9.045 | 0.205 | NA | NA | NA |

Created by EmpowerStats (www.empowerstats.com) and R on 2025-10-07
